# 4-[(3-Formyl-4-hydroxy­phen­yl)diazen­yl]-*N*-(pyrimidin-2-yl)benzene­sulfonamide

**DOI:** 10.1107/S1600536808024239

**Published:** 2008-08-06

**Authors:** Hoda El-Ghamry, Raafat Issa, Kamal El-Baradie, Keiko Isagai, Shigeyuki Masaoka, Ken Sakai

**Affiliations:** a Department of Chemistry, Faculty of Science, Tanta University, Tanta, Egypt; b Department of Chemistry, Faculty of Science, Kyushu University, Hakozaki 6-10-1, Higashi-ku, Fukuoka 812-8581, Japan

## Abstract

The title mol­ecule, C_17_H_13_N_5_O_4_S, has a *trans* configuration with respect to the diazenyl (azo) group. The pyrimidine ring and the terminal benzene ring are inclined at angles of 89.38 (4) and 1.6 (6)°, respectively, with respect to the central benzene ring. The conformation of the mol­ecule is in part stabilized by an intra­molecular O—H⋯O hydrogen bond. In the crystal structure, mol­ecules related through inversion centers form hydrogen-bonded dimers involving the sulfon­amide N—H group and the N atom of the pyrimidine ring.

## Related literature

For related literature, see: Gaber *et al.* (2008 ▶ and references therein); Kakoti *et al.* (1993 ▶); La Roche & Co (1967*a*,*b*); Misra *et al.* (1998 ▶); Mubarak *et al.* (2007 ▶); Nagaraja *et al.* (2002 ▶); Santra & Lahiri (1997 ▶); Vaichulis (1977 ▶). For bond-length data, see: Allen *et al.* (1987 ▶).
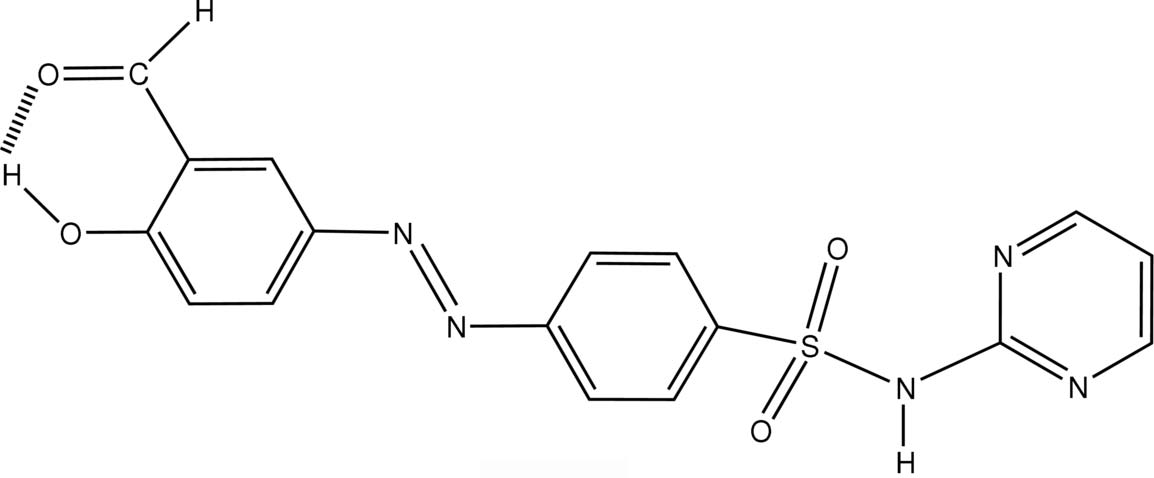


## Experimental

### 

#### Crystal data

C_17_H_13_N_5_O_4_S
*M* *_r_* = 383.39
Monoclinic, 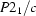

*a* = 18.579 (2) Å
*b* = 5.7731 (7) Å
*c* = 17.372 (2) Å
β = 115.99 (1)°
*V* = 1674.73 Å^3^
*Z* = 4
Mo *K*α radiation
μ = 0.23 mm^−1^
*T* = 100 (2) K
0.30 × 0.20 × 0.10 mm

#### Data collection

Bruker SMART APEXII CCD diffractometer
Absorption correction: multi-scan (*SADABS*; Sheldrick, 1996 ▶) *T*_min_ = 0.934, *T*_max_ = 0.977
17304 measured reflections
3560 independent reflections
3277 reflections with *I* > 2σ(*I*)
*R*_int_ = 0.017

#### Refinement

*R*[*F* ^2^ > 2σ(*F* ^2^)] = 0.034
*wR*(*F* ^2^) = 0.097
*S* = 1.05
3560 reflections
245 parameters
H-atom parameters constrained
Δρ_max_ = 0.42 e Å^−3^
Δρ_min_ = −0.37 e Å^−3^

### 

Data collection: *APEX2* (Bruker, 2006 ▶); cell refinement: *SAINT* (Bruker, 2004 ▶); data reduction: *SAINT*; program(s) used to solve structure: *SHELXS97* (Sheldrick, 2008 ▶); program(s) used to refine structure: *SHELXL97* (Sheldrick, 2008 ▶); molecular graphics: *KENX* (Sakai, 2004 ▶); software used to prepare material for publication: *SHELXL97*, *TEXSAN* (Molecular Structure Corporation, 2001 ▶), *KENX* and *ORTEPII* (Johnson, 1976 ▶).

## Supplementary Material

Crystal structure: contains datablocks global, I. DOI: 10.1107/S1600536808024239/lh2668sup1.cif

Structure factors: contains datablocks I. DOI: 10.1107/S1600536808024239/lh2668Isup2.hkl

Additional supplementary materials:  crystallographic information; 3D view; checkCIF report

## Figures and Tables

**Table 1:** Hydrogen-bond geometry (Å, °)

*D*—H⋯*A*	*D*—H	H⋯*A*	*D*⋯*A*	*D*—H⋯*A*
O2—H1⋯O1	0.84	1.90	2.6269 (17)	145
N3—H10⋯N5^i^	0.88	1.98	2.8574 (17)	179

Symmetry code: (i) 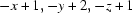.

**Fig. 1.**
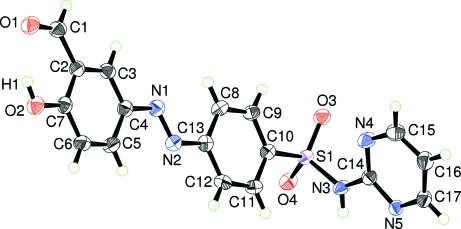
The molecular structure of (I) showing the atom-labeling scheme. Displacement ellipsoids are drawn at the 50% probability level.

**Fig. 2.**
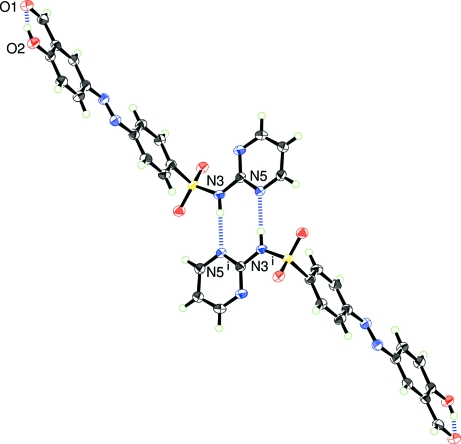
Part of the crystal structure showing a pair of molecules [symmetry code: (i)1 - x, 2 - y, 1 - z] stabilized by intra- and intermolecular hydrogen bonds (dashed lines).

## supplementary crystallographic information

### Comment

Sulfa-drugs are widely used in the treatment of infections, especially for patients intolerant to antibiotics (Nagaraja *et al.*, 2002). The vast commercial success of these medical agents has made the chemistry of sulfonamides become a major area of research and an important branch of commercial importance in pharmaceutical sciences (Nagaraja *et al.*, 2002). Heterocyclic azo compounds are also considered very important class of compounds. The importance of these compounds may stem from its biological activity and analytical investigations (Gaber *et al.*, 2008). Also, it is well known that heterocyclic azo compounds have been used to establish the low oxidation states of different metal ions (Kakoti *et al.*, 1993; Santra & Lahiri, 1997; Misra *et al.*, 1998). What appears more important is that sulfonamide and azo-sulfonamide derivatives have been found to be biologically versatile anticancer (La Roche & Co, 1967a,b) and antitubercular (Vaichulis, 1977) drugs.

In the title compound (I), C_17_H_13_N_5_O_4_S, the two aromatic groups attached to the azo double bond are oriented in a *trans* fashion. All the bond lengths are found to be within the normal values (Allen *et al.*, 1987). An intramolecular hydrogen bond is formed between the O—H (hydroxyl) group and the oxygen atom of the carbonyl group (O2—H1···O1; Table 1), stabilizing the coformation of the molecule. Moreover, the molecule is further correlated with an adjacent molecule through an inversion center, where the intermolecular interaction is stabilized with two hydrogen bonds formed between the *N*—*H*(sulfonamide) group and the nitrogen atom of pyrimidine ring, N3—H···N5^i^ hydrogen bond [symmetry code: (i) 1 - *x*, 2 - *y*, 1 - *z*] (see Fig. 2 and Table 1). There is an unusually short intermolecular contact between the C (carbonyl) and the O (sulfonamide) atoms, *i.e.*, C1···O3^ii^ = 2.91 (2) Å [symmetry code: (ii) 2 - *x*, 1/2 + *y*, 3/2 - *z*]. Although the rotation about the C10—S1 bond axis is essentially allowed, the rotation at this geometry is fixed as a result of strong intermolecular hydrogen bonds formed at the peripheral pyrimidine attached to the sulfonamide unit. This must be the major cause of the short contact found for one of the sulfonamide oxygen atoms, *i.e.*, O3.

The pyrimidine ring and both the central and terminal benzene rings are found to have a planar geometry (r.m.s deviations are 0.0136, 0.0106 and 0.0225 Å, respectively). Both the pyrimidine and the terminal phenyl rings are canted with respect to the central phenyl ring at angle of 89.38 (4) and 1.6 (6)°, respectively.

### Experimental

Compound (I) was prepared by the previously reported method in the literature (Mubarak *et al.*, 2007) in which 2.5 g m (0.01 mol) of sulfadiazine was dissolved in 25 ml of distilled water containing 2.5 ml (12*M*, 2.5 ml, 0.03 mol) of conc. HCl. The resulting solution was then cooled under stirring to 273 K. A cold solution containing 0.69 g m (0.01 mol) of sodium nitrite was drop wisely added to the previous solution to form the diazonium chloride. This solution was then added to a solution containing 1.06 ml of salicylaldehyde (0.01 mol) dissolved in 10 ml of water containing 0.4 g m (0.01 mol) of NaOH. The orange dye, which separated out, was kept in an ice bath under stirring for 30 minutes. The precipitate was then filtered off, washed by distilled water, and finally air_dried (yield: 80%). Analysis calculated for C_17_H_13_N_5_O_4_S: C, 53.26; H, 3.39; N, 18.27; S, 8.35% found: C, 53.44; H, 3.34; N, 18.38; S, 8.25%. IR (ν, cm^-1^): 3424(w), 3358(w), 3082(w), 3041(w), 2944(w), 2814(w), 2727(w), 1652(*s*), 1621(*m*), 1581(*s*), 1496(*s*), 1443(*s*), 1410(*s*), 1384(*m*), 1339(*s*), 1305(*m*), 1289(*s*), 1269(*m*), 1206(*m*), 1166(*s*), 1154(*s*), 1141(w), 1001(*m*), 952(*s*), 902(*m*), 840(*s*), 797(*s*), 772(w), 750(*s*), 729(*s*), 669(*s*), 640(*s*), 606(*s*), 582(*s*), 564(*s*), 540(w), 531(w), 521(*m*), 509(*m*), 455(*s*), 432(*m*), 424(w), 403(*m*). A good crystal suitable for *x*-ray measurement was prepared by vapour diffusion method in which compound (I) was dissolved in the least possible amount of DMF and then the solution was left at room temperature in the presence of water pool outside. The outside water slowly diffused by vapour and gradually mixed with DMF of the dye. After 7 days, dark orange crystals suitable for X-ray diffraction analysis was separated out.

### Refinement

All H atoms were placed in idealized positions, (C—H = 0.95 Å, O—H = 0.84 Å, and N—H = 0.88 Å), and included in the refinement in a riding-model approximation, with *U*_iso_(H) = 1.2Ueq (C and N) and *U*_iso_(H) = 1.5Ueq (O). In the final difference Fourier map, the highest peak was located 0.83 Å from atom C10. The deepest hole was located 0.64 Å from atom S1.

### Crystal data

**Table:**

C_17_H_13_N_5_O_4_S	*F*_000_ = 792
*M**_r_* = 383.39	? # Insert any comments here.
Monoclinic, *P*2_1_/*c*	*D*_x_ = 1.521 Mg m^−^^3^
Hall symbol: -P 2ybc	Mo *K*α radiation λ = 0.71073 Å
*a* = 18.579 (2) Å	Cell parameters from 9950 reflections
*b* = 5.7731 (7) Å	θ = 2.4–28.3º
*c* = 17.372 (2) Å	µ = 0.23 mm^−^^1^
β = 115.99 (1)º	*T* = 100 (2) K
*V* = 1674.73 Å^3^	Block, dark orange
*Z* = 4	0.30 × 0.20 × 0.10 mm

### Data collection

**Table:**

Bruker SMART APEX CCD diffractometer	3560 independent reflections
Radiation source: fine-focus sealed tube	3277 reflections with *I* > 2σ(*I*)
Monochromator: graphite	*R*_int_ = 0.017
*T* = 100(2) K	θ_max_ = 26.7º
φ and ω scans	θ_min_ = 2.4º
Absorption correction: multi-scan(SADABS; Sheldrick, 1996)	*h* = −23→23
*T*_min_ = 0.934, *T*_max_ = 0.977	*k* = −7→7
17304 measured reflections	*l* = −21→21

### Refinement

**Table:**

Refinement on *F*^2^	Hydrogen site location: inferred from neighbouring sites
Least-squares matrix: full	H-atom parameters constrained
*R*[*F*^2^ > 2σ(*F*^2^)] = 0.034	*w* = 1/[σ^2^(*F*_o_^2^) + (0.0513*P*)^2^ + 0.9337*P*] where *P* = (*F*_o_^2^ + 2*F*_c_^2^)/3
*wR*(*F*^2^) = 0.097	(Δ/σ)_max_ = 0.001
*S* = 1.05	Δρ_max_ = 0.42 e Å^−^^3^
3560 reflections	Δρ_min_ = −0.37 e Å^−^^3^
245 parameters	Extinction correction: none
Secondary atom site location: difference Fourier map	

### Special details

**Table:**

Experimental. The first 50 frames were rescanned at the end of data collection to evaluate any possible decay phenomenon. Since it was judged to be negligible, no decay correction was applied to the data.
Geometry. All e.s.d.'s (except the e.s.d. in the dihedral angle between two l.s. planes) are estimated using the full covariance matrix. The cell e.s.d.'s are taken into account individually in the estimation of e.s.d.'s in distances, angles and torsion angles; correlations between e.s.d.'s in cell parameters are only used when they are defined by crystal symmetry. An approximate (isotropic) treatment of cell e.s.d.'s is used for estimating e.s.d.'s involving l.s. planes.Least-squares planes (*x*,*y*,*z* in crystal coordinates) and deviations from them (* indicates atom used to define plane)13.7836 (0.0041) *x* + 2.4391 (0.0022) *y* - 13.7785 (0.0036) *z* = 4.2437 (0.0052)* 0.0258 (0.0010) O1 * -0.0309 (0.0010) O2 * 0.0133 (0.0012) C1 * -0.0188 (0.0013) C2 * -0.0067 (0.0012) C3 * 0.0010 (0.0012) C4 * 0.0430 (0.0013) C5 * 0.0139 (0.0012) C6 * -0.0207 (0.0013) C7 * -0.0198 (0.0009) N1 - 0.1255 (0.0017) N2 - 0.2396 (0.0024) C9 - 0.2576 (0.0024) C10 - 0.2407 (0.0024) C11 - 0.2117 (0.0022) C12 - 0.1607 (0.0019) C13Rms deviation of fitted atoms = 0.022513.5655 (0.0045) *x* + 2.3850 (0.0032) *y* - 14.0573 (0.0043) *z* = 3.6030 (0.0035)Angle to previous plane (with approximate e.s.d.) = 1.60 (0.06)* 0.0132 (0.0011) N2 * -0.0021 (0.0008) C9 * -0.0021 (0.0012) C10 * -0.0053 (0.0012) C11 * -0.0149 (0.0012) C12 * 0.0178 (0.0013) C13 * -0.0066 (0.0004) O1 - 0.0637 (0.0021) O2 0.0152 (0.0018) C1 0.0043 (0.0016) C2 0.0555 (0.0016) C3 0.0836 (0.0016) C4 0.1066 (0.0021) C5 0.0390 (0.0023) C6 - 0.0162 (0.0020) C7 0.1025 (0.0013) N1Rms deviation of fitted atoms = 0.01062.8895 (0.0099) *x* + 3.8287 (0.0026) *y* + 10.2469 (0.0065) *z* = 10.3651 (0.0036)Angle to previous plane (with approximate e.s.d.) = 89.38 (0.04)* -0.0192 (0.0009) N3 * 0.0179 (0.0011) N4 * 0.0136 (0.0010) N5 * 0.0038 (0.0012) C14 * -0.0009 (0.0012) C15 * -0.0197 (0.0013) C16 * 0.0045 (0.0011) C17Rms deviation of fitted atoms = 0.0136
Refinement. Refinement of *F*^2^ against ALL reflections. The weighted *R*-factor *wR* and goodness of fit *S* are based on *F*^2^, conventional *R*-factors *R* are based on *F*, with *F* set to zero for negative *F*^2^. The threshold expression of *F*^2^ > σ(*F*^2^) is used only for calculating *R*-factors(gt) *etc*. and is not relevant to the choice of reflections for refinement. *R*-factors based on *F*^2^ are statistically about twice as large as those based on *F*, and *R*- factors based on ALL data will be even larger.

### Fractional atomic coordinates and isotropic or equivalent isotropic displacement parameters (Å^2^)

**Table:**

	*x*	*y*	*z*	*U*_iso_*/*U*_eq_	
S1	0.67904 (2)	0.77281 (6)	0.52372 (2)	0.02455 (11)	
O1	1.32533 (7)	0.9734 (2)	1.18827 (7)	0.0366 (3)	
N5	0.49729 (7)	0.7981 (2)	0.57442 (7)	0.0246 (3)	
O4	0.67616 (6)	0.9243 (2)	0.45700 (6)	0.0301 (2)	
O3	0.68746 (6)	0.52911 (19)	0.51537 (7)	0.0297 (2)	
C17	0.46694 (9)	0.6915 (3)	0.62192 (9)	0.0289 (3)	
H13	0.4169	0.7418	0.6183	0.035*	
N2	0.94619 (7)	1.1123 (2)	0.84456 (8)	0.0282 (3)	
N3	0.59631 (7)	0.8311 (2)	0.53095 (8)	0.0280 (3)	
H10	0.5668	0.9446	0.4987	0.034*	
N1	1.00856 (7)	0.9914 (2)	0.87789 (8)	0.0280 (3)	
O2	1.24471 (7)	1.3545 (2)	1.17919 (7)	0.0332 (3)	
H1	1.2835	1.2624	1.2013	0.050*	
C15	0.57921 (10)	0.4485 (3)	0.68056 (10)	0.0357 (4)	
H11	0.6079	0.3262	0.7181	0.043*	
C16	0.50602 (9)	0.5108 (3)	0.67607 (10)	0.0338 (3)	
H12	0.4835	0.4332	0.7086	0.041*	
C1	1.26947 (9)	0.9081 (3)	1.12173 (10)	0.0310 (3)	
H2	1.2736	0.7616	1.0991	0.037*	
C5	1.06241 (9)	1.3131 (3)	0.98413 (10)	0.0297 (3)	
H4	1.0171	1.4067	0.9518	0.036*	
C6	1.12111 (9)	1.3967 (3)	1.05977 (10)	0.0299 (3)	
H5	1.1155	1.5451	1.0802	0.036*	
C12	0.81694 (9)	1.1545 (3)	0.72898 (9)	0.0288 (3)	
H9	0.8145	1.2998	0.7534	0.035*	
C11	0.75308 (9)	1.0818 (3)	0.65434 (9)	0.0286 (3)	
H8	0.7074	1.1777	0.6262	0.034*	
C8	0.88818 (9)	0.7981 (3)	0.73479 (9)	0.0279 (3)	
H6	0.9343	0.7034	0.7621	0.034*	
C7	1.18899 (9)	1.2628 (3)	1.10648 (9)	0.0269 (3)	
C4	1.06845 (9)	1.0915 (3)	0.95400 (9)	0.0265 (3)	
C3	1.13632 (9)	0.9607 (3)	0.99930 (9)	0.0266 (3)	
H3	1.1417	0.8129	0.9783	0.032*	
C9	0.82375 (9)	0.7222 (3)	0.66129 (9)	0.0264 (3)	
H7	0.8249	0.5737	0.6383	0.032*	
C13	0.88463 (9)	1.0158 (3)	0.76846 (9)	0.0266 (3)	
C2	1.19719 (9)	1.0441 (3)	1.07584 (9)	0.0266 (3)	
N4	0.61192 (8)	0.5519 (2)	0.63453 (8)	0.0328 (3)	
C14	0.56803 (8)	0.7196 (2)	0.58287 (9)	0.0246 (3)	
C10	0.75726 (8)	0.8652 (3)	0.62140 (9)	0.0242 (3)	

### Atomic displacement parameters (Å^2^)

**Table:**

	*U*^11^	*U*^22^	*U*^33^	*U*^12^	*U*^13^	*U*^23^
S1	0.02184 (18)	0.0280 (2)	0.02274 (19)	0.00350 (13)	0.00873 (14)	0.00169 (13)
O1	0.0312 (6)	0.0394 (6)	0.0338 (6)	0.0059 (5)	0.0091 (5)	0.0096 (5)
N5	0.0208 (6)	0.0265 (6)	0.0224 (6)	0.0000 (5)	0.0057 (5)	0.0021 (5)
O4	0.0290 (5)	0.0365 (6)	0.0256 (5)	0.0041 (4)	0.0127 (4)	0.0044 (4)
O3	0.0266 (5)	0.0294 (6)	0.0289 (5)	0.0017 (4)	0.0083 (4)	−0.0033 (4)
C17	0.0238 (7)	0.0335 (8)	0.0269 (7)	−0.0013 (6)	0.0090 (6)	0.0028 (6)
N2	0.0279 (6)	0.0288 (6)	0.0271 (6)	0.0006 (5)	0.0114 (5)	−0.0006 (5)
N3	0.0231 (6)	0.0318 (7)	0.0284 (6)	0.0076 (5)	0.0107 (5)	0.0102 (5)
N1	0.0277 (6)	0.0293 (6)	0.0275 (6)	0.0005 (5)	0.0127 (5)	0.0003 (5)
O2	0.0292 (6)	0.0361 (6)	0.0280 (5)	0.0013 (5)	0.0066 (4)	−0.0008 (5)
C15	0.0342 (8)	0.0351 (8)	0.0319 (8)	0.0044 (7)	0.0091 (6)	0.0127 (7)
C16	0.0312 (8)	0.0368 (8)	0.0310 (7)	−0.0031 (6)	0.0115 (6)	0.0091 (6)
C1	0.0311 (7)	0.0297 (8)	0.0333 (8)	0.0039 (6)	0.0150 (6)	0.0073 (6)
C5	0.0255 (7)	0.0300 (8)	0.0311 (8)	0.0052 (6)	0.0100 (6)	0.0016 (6)
C6	0.0301 (7)	0.0261 (7)	0.0323 (8)	0.0024 (6)	0.0127 (6)	−0.0017 (6)
C12	0.0341 (8)	0.0228 (7)	0.0294 (7)	0.0023 (6)	0.0139 (6)	−0.0011 (6)
C11	0.0295 (7)	0.0258 (7)	0.0289 (7)	0.0065 (6)	0.0114 (6)	0.0034 (6)
C8	0.0245 (7)	0.0319 (8)	0.0267 (7)	0.0056 (6)	0.0105 (6)	0.0004 (6)
C7	0.0256 (7)	0.0298 (7)	0.0257 (7)	−0.0016 (6)	0.0116 (6)	0.0022 (6)
C4	0.0264 (7)	0.0287 (7)	0.0256 (7)	−0.0005 (6)	0.0126 (6)	0.0003 (6)
C3	0.0297 (7)	0.0240 (7)	0.0296 (7)	0.0006 (6)	0.0162 (6)	0.0016 (6)
C9	0.0272 (7)	0.0264 (7)	0.0266 (7)	0.0044 (6)	0.0129 (6)	−0.0010 (6)
C13	0.0272 (7)	0.0289 (7)	0.0243 (7)	−0.0008 (6)	0.0118 (6)	0.0002 (6)
C2	0.0265 (7)	0.0272 (7)	0.0280 (7)	0.0011 (6)	0.0138 (6)	0.0057 (6)
N4	0.0275 (6)	0.0359 (7)	0.0309 (7)	0.0074 (5)	0.0091 (5)	0.0113 (6)
C14	0.0220 (6)	0.0261 (7)	0.0220 (6)	0.0002 (5)	0.0064 (5)	0.0011 (5)
C10	0.0236 (7)	0.0276 (7)	0.0214 (6)	0.0013 (5)	0.0099 (5)	0.0012 (5)

### Geometric parameters (Å, °)

**Table:**

S1—O3	1.4299 (11)	C11—C10	1.391 (2)
S1—O4	1.4343 (11)	C8—C9	1.384 (2)
S1—N3	1.6311 (12)	C8—C13	1.400 (2)
S1—C10	1.7647 (15)	C7—C2	1.404 (2)
O1—C1	1.226 (2)	C4—C3	1.382 (2)
N5—C14	1.3362 (19)	C3—C2	1.400 (2)
N5—C17	1.3362 (19)	C9—C10	1.392 (2)
C17—C16	1.379 (2)	C1—O3i	2.9106 (19)
N2—N1	1.2558 (18)	C5—H4	0.9500
N2—C13	1.4285 (19)	N3—H10	0.8800
N3—C14	1.3857 (19)	C1—H2	0.9500
N4—C14	1.3289 (19)	C6—H5	0.9500
N1—C4	1.4250 (19)	C8—H6	0.9500
O2—C7	1.3420 (18)	C15—H11	0.9500
C15—N4	1.338 (2)	C16—H12	0.9500
C15—C16	1.375 (2)	C12—H9	0.9500
C1—C2	1.455 (2)	O2—H1	0.8400
C5—C6	1.376 (2)	C11—H8	0.9500
C5—C4	1.405 (2)	C3—H3	0.9500
C6—C7	1.396 (2)	C17—H13	0.9500
C12—C11	1.384 (2)	C9—H7	0.9500
C12—C13	1.392 (2)		
			
O3—S1—O4	119.02 (7)	C13—C12—H9	119.8
O3—S1—N3	111.08 (7)	C12—C11—C10	118.57 (13)
O4—S1—N3	103.51 (6)	C12—C11—H8	120.7
O3—S1—C10	108.31 (7)	C10—C11—H8	120.7
O4—S1—C10	108.42 (7)	C9—C8—C13	119.32 (13)
N3—S1—C10	105.68 (7)	C9—C8—H6	120.3
C14—N5—C17	115.89 (13)	C13—C8—H6	120.3
N5—C17—C16	122.09 (14)	O2—C7—C6	117.16 (14)
N5—C17—H13	119.0	O2—C7—C2	122.97 (13)
C16—C17—H13	119.0	C6—C7—C2	119.87 (14)
N1—N2—C13	114.37 (13)	C3—C4—C5	119.16 (14)
C14—N3—S1	126.38 (10)	C3—C4—N1	116.95 (13)
C14—N3—H10	116.8	C5—C4—N1	123.89 (13)
S1—N3—H10	116.8	C4—C3—C2	120.56 (14)
N2—N1—C4	112.94 (13)	C4—C3—H3	119.7
C7—O2—H1	109.5	C2—C3—H3	119.7
N4—C15—C16	123.09 (15)	C8—C9—C10	119.44 (14)
N4—C15—H11	118.5	C8—C9—H7	120.3
C16—C15—H11	118.5	C10—C9—H7	120.3
C15—C16—C17	116.64 (14)	C12—C13—C8	120.55 (14)
C15—C16—H12	121.7	C12—C13—N2	114.57 (13)
C17—C16—H12	121.7	C8—C13—N2	124.86 (13)
O1—C1—C2	123.15 (15)	C3—C2—C7	119.50 (13)
O1—C1—H2	118.4	C3—C2—C1	120.14 (14)
C2—C1—H2	118.4	C7—C2—C1	120.34 (14)
C6—C5—C4	121.07 (14)	C14—N4—C15	115.06 (13)
C6—C5—H4	119.5	N4—C14—N5	127.19 (14)
C4—C5—H4	119.5	N4—C14—N3	118.86 (13)
C5—C6—C7	119.78 (14)	N5—C14—N3	113.95 (12)
C5—C6—H5	120.1	C11—C10—C9	121.71 (13)
C7—C6—H5	120.1	C11—C10—S1	119.74 (11)
C11—C12—C13	120.37 (14)	C9—C10—S1	118.47 (11)
C11—C12—H9	119.8		
			
C14—N5—C17—C16	−0.3 (2)	C4—C3—C2—C1	−178.17 (13)
O3—S1—N3—C14	46.71 (15)	O2—C7—C2—C3	−179.94 (13)
O4—S1—N3—C14	175.57 (13)	C6—C7—C2—C3	−1.2 (2)
C10—S1—N3—C14	−70.55 (14)	O2—C7—C2—C1	−1.9 (2)
C13—N2—N1—C4	−179.50 (12)	C6—C7—C2—C1	176.90 (14)
N4—C15—C16—C17	−1.1 (3)	O1—C1—C2—C3	178.82 (14)
N5—C17—C16—C15	1.6 (2)	O1—C1—C2—C7	0.8 (2)
C4—C5—C6—C7	1.8 (2)	C16—C15—N4—C14	−0.6 (2)
C13—C12—C11—C10	1.9 (2)	C15—N4—C14—N5	2.2 (2)
C5—C6—C7—O2	179.17 (13)	C15—N4—C14—N3	−178.08 (14)
C5—C6—C7—C2	0.3 (2)	C17—N5—C14—N4	−1.7 (2)
C6—C5—C4—C3	−3.0 (2)	C17—N5—C14—N3	178.51 (13)
C6—C5—C4—N1	177.69 (14)	S1—N3—C14—N4	2.0 (2)
N2—N1—C4—C3	174.33 (13)	S1—N3—C14—N5	−178.19 (11)
N2—N1—C4—C5	−6.4 (2)	C12—C11—C10—C9	−0.3 (2)
C5—C4—C3—C2	2.2 (2)	C12—C11—C10—S1	−177.03 (11)
N1—C4—C3—C2	−178.51 (12)	C8—C9—C10—C11	−1.2 (2)
C13—C8—C9—C10	1.1 (2)	C8—C9—C10—S1	175.57 (11)
C11—C12—C13—C8	−2.0 (2)	O3—S1—C10—C11	−165.58 (11)
C11—C12—C13—N2	179.57 (13)	O4—S1—C10—C11	63.96 (13)
C9—C8—C13—C12	0.4 (2)	N3—S1—C10—C11	−46.48 (13)
C9—C8—C13—N2	178.74 (14)	O3—S1—C10—C9	17.59 (14)
N1—N2—C13—C12	−177.01 (13)	O4—S1—C10—C9	−112.87 (12)
N1—N2—C13—C8	4.6 (2)	N3—S1—C10—C9	136.69 (12)
C4—C3—C2—C7	−0.1 (2)		

Symmetry codes: i.

### Hydrogen-bond geometry (Å, °)

**Table:**

*D*—H···*A*	*D*—H	H···*A*	*D*···*A*	*D*—H···*A*
O2—H1···O1	0.84	1.90	2.6269 (17)	145
N3—H10···N5^i^	0.88	1.98	2.8574 (17)	179

Symmetry codes: (i) −*x*+1, −*y*+2, −*z*+1.
